# Comparative Metagenomic Analysis of Two Alkaline Hot Springs of Madhya Pradesh, India and Deciphering the Extremophiles for Industrial Enzymes

**DOI:** 10.3389/fgene.2021.643423

**Published:** 2021-03-08

**Authors:** Kamlesh Choure, Shreyansh Parsai, Rhitu Kotoky, Arpit Srivastava, Anita Tilwari, Piyush Kant Rai, Abhishek Sharma, Piyush Pandey

**Affiliations:** ^1^Department of Biotechnology, AKS University, Satna, India; ^2^Department of Microbiology, Assam University, Silchar, India; ^3^Centre of Excellence in Biotechnology, Madhya Pradesh Council of Science and Technology, Bhopal, India; ^4^Amity Food and Agriculture Foundation, Amity University, Noida, India

**Keywords:** microbiome, Hotsprings, extremophiles, microbial diversity, industrial enzymes

## Abstract

Hot springs are considered to be a unique environment with extremophiles, that are sources of industrially important enzymes, and other biotechnological products. The objective of this study was to undertake, analyze, and characterize the microbiome of two major hot springs located in the state of Madhya Pradesh explicitly, Chhoti Anhoni (Hotspring 1), and Badi Anhoni (Hotspring 2) to find out the inhabitant microbial population, and their functional characteristics. The taxonomic analysis of the microbiome of the hot springs revealed the phylum Proteobacteria was the most abundant taxa in both the hot-springs, however, its abundance in hot-spring 1 (~88%) was more than the hot-spring 2 (~52%). The phylum Bacteroides (~10–22%) was found to be the second most abundant group in the hot-springs followed by Spirocheates (~2–11%), Firmicutes (~6–8%), Chloroflexi (1–5%), etc. The functional analysis of the microbiome revealed different features related to several functions including metabolism of organics and degradation of xenobiotic compounds. The functional analysis showed that most of the attributes of the microbiome was related to metabolism, followed by cellular processes and environmental information processing functions. The functional annotation of the microbiomes at KEGG level 3 annotated the sequences into 279 active features that showed variation in abundance between the hot spring samples, where hot-spring 1 was functionally more diverse. Interestingly, the abundance of functional genes from methanogenic bacteria, was higher in the hot-spring 2, which may be related to the relatively higher pH and temperature than Hotspring 1. The study showed the presence of different unassigned bacterial taxa with high abundance which indicates the potential of novel genera or phylotypes. Culturable isolates (28) were bio-prospected for industrially important enzymes including amylase, protease, lipase, gelatinase, pectinase, cellulase, lecithinase, and xylanase. Seven isolates (25%) had shown positive results for all the enzyme activities whereas 23 isolates (82%) produced Protease, 27 isolates (96%) produced lipase, 27 isolates produced amylase, 26 isolates (92%) produced cellulase, 19 isolates (67%) produced pectinase, 19 isolates (67%) could produce lecithinase, and 13 isolates (46%) produced gelatinase. The seven isolates, positive for all the enzymes were analyzed further for quantitative analysis and identified through molecular characterization.

## Introduction

Extremophilic microorganisms thrive in diverse and extreme conditions and constitute a major part of the biosphere (Mirete et al., 2016). The thermophiles and hyper-thermophiles live in high-temperature environments such as hot springs, though few of these can survive in co-existing, more than one extreme conditions, like acidic or alkaline hot springs. The accessibility of thermophiles to survive at high temperatures is related to their incredibly thermostable macromolecules present in them (Brock, 2001). These thermophilic microorganisms have been studied extensively for thermostable enzymes such as amylases, cellulases, chitinases, pectinases, xylanases, proteases, lipase, and DNA polymerases, etc. that has unique features of biotechnological processes (Singh et al., 2011).

Thermophilic microorganisms are an excellent source of thermostable enzymes and have been utilized in the greater part of industrial applications, for example, food, papers, pharmaceutical, cleansers, etc. (Schuler et al., 2017; Roy et al., 2020). Thermophilic microorganisms are also more stable than their mesophilic partners to natural solvents, cleansers, low and high pH, and other extreme conditions (Demirjian et al., 2001). Therefore, industrially important enzymes from thermophiles such as amylase (extracellular), protease (extracellular), lipase (extra/intracellular), gelatinase (extracellular), pectinase (extra/intracellular), cellulase (extra/intracellular), lecithinase (extracellular), and xylanase (extracellular) has been used extensively. Most of these enzymes are found to be optimally active at temperatures close to the host organism's optimal growth temperature. However, some of the extracellular and cell-bound hyperthermophilic enzymes were optimally active at temperatures above, sometimes far above than the host organism's optimum growth temperature (Vieille and Zeikus, 2001).

The Geological Survey of India has identified about 340 hot springs located in different parts of India, which are characterized by their orogenic activities (Chandrasekharam, 2005; Craig et al., 2013). All these hot springs have been classified and grouped into nine geothermal provinces based on their geo-tectonic setup that includes the Himalayas, Naga-Lushai province, Sohana, West coast, Andaman-Nicobar Islands, Cambay, Son-Narmada-Tapi (SONATA), Godavari, and Mahanadi valleys. Geothermal resources along Son-Narmada lineament viz. Choti and Badi Anhoni form the most promising resource base in central India (Shanker, 1986). The lineament is one of the most important lineaments/rifted structure of the sub-continent. It runs across the country in an almost East-West direction and has a long history of tectonic reactivation. It contains several known thermal spring areas, the most interesting one being those situated at Anhoni (Saxena et al., 2017). There are several hot springs situated in Madhya Pradesh at several locations like Anhoni in Chhindwara district, hot and boiling sulfur springs that flow along within the forest. Anhoni is particularly known for its 'boiling water *kund'* (*kund* means a small pond), Choti Anhoni near Pipariya, Badi Anhoni near Panchmarhi, Chavalpani at Pachmarhi, Anhoni Samoni (it is different from the aforementioned Anhoni springs), Babeha hot spring is in the Mandla district, and Dhuni Pani, Amarkantak. The alkaline hot springs have pH more than seven and can range from 8.5 to 12. Other alkaline hot-springs have also been studied, from other parts of world, such as the Great Rift Valley in northeastern Africa, which has been characterized to have high levels of carbonates, chlorides, and silica compounds (Jones et al., 1998). The organisms surviving in such alkaline hot springs acquire necessary adaptations. The bacteria present in such environments are either alkaliphilic or alkalitolerant, that are known as alkalithermophilic bacteria, and these organisms have enzymes to support their growth and survival in such extreme conditions. These alkalithermophiles are often reported to be chemolithoautotrophic (Sorokin and Kuenen, 2005).

Several studies have been done to analyze the microbial diversity of different hot-springs around the globe. The microorganisms growing in different ecological zones (e.g., hot springs and deep-sea) can be categorized into moderate thermophiles (growth optimum, 50–60°C), extreme thermophiles (growth optimum, 60–80°C), and hyperthermophiles (growth optimum, 80–110°C) (Gupta et al., 2014). The natural habitats of the thermophiles include continental solfataras, deep geothermally heated oil-containing stratifications, shallow marine, and deep-sea hot sediments, and hydrothermal vents. The hyperthermophiles have also been isolated from hot industrial environments. These hyperthermophiles with the highest growth temperatures are members of the genera Pyrobaculum, Pyrodictium, Pyrococcus, and Melanopyrus belonging to Archaea. However, the isolation and growth of pure cultures of novel hyperthermophiles has been a challenge, which mostly remains unculturable, and may be assessed using metagenomics and next-generation sequencing technologies (López-López et al., 2013).

The present study was taken to analyze the taxonomical and functional diversity of the microbiome of two alkaline hot-springs with idea to analyze the genetic pool of thermophilic microorganisms as a source of industrially important enzymes. This research describes the insights of their microbial diversity, including strategies followed by enzyme screening and quantifications.

## Materials and Methods

### Collection of Samples

Water samples were collected from the Choti and Badi Anhoni Hot Springs (22.65°N latitude and 78.36°E longitude) situated in Panchmari, Madhya Pradesh (India). Physiological parameters of water samples were measured on-site using HANNA HI2300 EC/TDS/NaCl multi-probe system according to the manual. The sample of Choti Anhoni and Badi Anhoni are designated as Hotspring 1 and Hotspring 2, respectively.

### Isolation and Characterization of Thermophilic Bacteria

The thermophilic bacteria from the water samples were isolated according to methods described by Adiguzel et al. (2009), through the serial dilution method. Thermophilic bacteria were isolated and cultured on Nutrient agar plates, the pH of the medium was adjusted to 7.0 before autoclaving and then incubated at 45°C for 24–48 h (Sikdar et al., 2015). Isolation of pure cultures was done using the streak plate method and the cultures were stored for enzyme screening analysis.

The selected bacterial isolates (positive for all the enzyme activity, as tested) were subjected to identification based on 16S rDNA gene sequencing. DNA isolated from the bacterial isolates was directly used for PCR amplification of 16S rRNA gene using 1492R (5′CGGTTACCTTGTTACGACTT3′) and 27F (5′AGAGTTTGATCMTGGCTCAG3′) universal primers. The sequence obtains after sequencing were used for the *in silico* study to obtain the highest similarity using online web server nucleotide BLASTN based on the BLAST alignment.

### Analysis of Extracellular Enzymes

The isolates were analyzed for different extracellular enzymes of industrial importance like protease, lipase, amylase, xylanase, cellulase, pectinase, lecithinase, and gelatinase. The screening for protease activity was performed as described (Bragger et al., 1989), on skim milk agar containing 8 g/L nutrient broth, 10 g/L skim milk, and 17 g/L agar, then incubated for 36 h at 45°C. The presence of protease activity was confirmed by the appearance of clear zones around the well indicating degradation of casein milk.

The lipase activity of the isolates was performed according to the method described by Haba et al. (2000), on a medium containing 8 g/L nutrient broth, 0.25 g/L CaCl2.2H2O, 9 g/L agar dissolved in 500 mL deionized water, and 5 mL of Tween 20 dissolved in 500 mL deionized water autoclaved separately and add to the medium, then the medium with the cultures incubated for 2 days at 45°C. Clear zones that occur around the colonies indicated the presence of lipase activity. The screening of the amylase activity was performed as described (Bragger et al., 1989), on a medium containing 1 g/L yeast extract, 5 g/L soluble starch, and 17 g/L agar. Ingredients were dissolved in deionized water and sterilized by autoclaved and incubated for 1–2 days at 45°C. The presence of amylase activity was confirmed by the appearance of a clear halo around the well after the color with iodine.

The xylanase activity was performed according to the method described by Bragger et al. (1989), on a medium containing 1 g/L yeast extract, 5 g/L xylans, and 17 g/L agar, which was incubated for 3–4 days at 45°C. The activity of the xylanase enzyme was confirmed by the appearance of a clear zone around the tested strain following the staining with Congo Red. Similarly, the activity of cellulase was performed according to the method described by Bragger et al. (1989), on a medium containing 1 g/L yeast extract, 5 g/L carboxymethyl cellulose (CMC) salt, and 17 g/L agar then incubated for 3–4 days at 45°C. Cellulase activity resulted in the appearance of a clear zone around the tested strain after treatment with iodine. Identification of bacterial isolates displaying pectinase activity was performed according to Bragger et al. (1989), on a medium containing 1 g/L yeast extract, 2 g/L ammonium sulfate, 6 g/L Na2HPO4, 3 g/L KH_2_PO_4_, 5 g/L pectins, and 17 g/L agar. Ingredients were dissolved in deionized water and sterilized by autoclaved at 121°C for 15 min and incubated for 3–4 days at 45°C. Colonies with clear zones indicated pectinase activity.

Lecithinase production was tested on a modified medium as described previously (Oladipo et al., 2008). Lecithinase was detected according to the standard method (Sharaf et al., 2014), in which 1 ml of each bacterial culture, having cell density of 6 × 10^8^ CFU/ml was inoculated into test tubes containing corn millet broth and incubated for 24 h at 37°C. After incubation, the cultures were centrifuged at 2500 rpm for 15 min to obtain a cell-free filtrate, and 100 μl of the filtrate was transferred into 10-mm wells made centrally in the egg-yolk agar plates and incubated for 24 h at 37°C. Opaque zones were measured as indicators of lecithinase production. Gelatinase production was detected by stab inoculating the test strain on nutrient agar supplemented with 3% gelatin kept at 37°C for 24 h followed by refrigeration at 4°C for 30 min. Liquefaction of gelatin was taken positive (Betty et al., 2007).

### Quantitative Estimation of Enzyme Activities

The isolates positive for all the tested enzymatic production were further analyzed for the quantitative estimation of enzyme activities at ambient temperatures and pH (of respective site, as described in **Table 2**) i.e., 55/65°C and 8.5/9.5, respectively. To determine the cellulase activity, colorimetric assay by DNS (Dinitro salicylic acid) method was used (Miller, xbib1995). Samples were subjected to incubation for 30 min with CMC (Carboxymethyl cellulose) as substrate followed by the addition of DNS and boiled for 6–7 min, and absorbance was taken at 540 nm. Similarly for amylase activity, 1 ml of enzyme solution was incubated with substrate solution, containing 1% (w/v) (1ml) soluble starch at 55/65°C for 30 min followed by the addition of DNS to stop the reaction and kept at boiling water bath for 10 min (Bernfeld, 1955). Lipase enzyme activity was performed by using 1% tributyrin in basal salt media. P-Nitrophenol dodecanote was used as a substrate to determine lipase activity. The reaction mixture containing enzyme solution and P-Nitrophenol dodecanote was incubated for 30 min at 55/65°C. For the protease activity, casein is used as a substrate, and the reaction mixture was composed of 2.5 ml of the substrate and 1 ml cell-free extract enzyme solution followed by the incubation at 55/65°C for 30 min. Trichloroacetic acid is used for the termination of the reaction. Gelatinase activity was measured by using gelatin as a substrate, where 0.2 ml of 50% Trichloroacetic acid was used to terminate the reaction. The Lecithinase activity was performed on 10 ml 50% egg yolk in basal salt media. The activity was measured by using the method described by McLaughlin and (McLaughlin and Weiss, 1996). All the observations were recorded by taking absorbance at 540 nm using a spectrophotometer.

### Microbiome Analysis of the Hot-Springs

The water samples from the hot springs were collected and analyzed for the microbiomes through metagenome analysis of the hypervariable V3–V4 region. The DNA was obtained from the water samples using the Nucleospin DNA kit. The amplicon libraries were prepared using the Nextera Index kit as per the 16S metagenomic sequencing library preparation protocol. For this, 16S rDNA specific primers were used for bacterial V3–V4. The libraries were sequenced on MiSeq using a 2×300 bp paired-end manner. The amplicons with the Illumina adapters were amplified by using i5 and i7 primers and purified by AMPureXP beads and quantified using a Qubit fluorometer. After that, the libraries were loaded onto Miseq at the appropriate concentration for cluster generation and sequencing (Kotoky and Pandey, 2020).

Quality Control was performed using the online FastQC tool v 0.11.7. Read quality was good with an average of more than 200,000 (2 lakh) reads per sample and a read length of 300 bp. High-quality reads were taken for further analysis. The fastq-Join tool was used to convert the overlapping paired-end reads into a consensus sequence of the V3–V4 region. It finds the overlap for each pair and combines them into a single read. In the Pre-processing step, Chimeric sequences were filtered out using the parameter reference_chimera_detection default implemented in the QIIME tool. OTU Picking and Taxonomic classification were performed using the UCLUST method in the QIIME. Reads from all samples were pooled and clustered into Operational Taxonomic Unit (OTU) based sequence similarity of >=97% with help of UCLUST method with reference to green gene database. Finally, 485 OTUs were identified at the species level.

After sequencing the paired-end sequences were analyzed as described by Kotoky and Pandey (2020). The Quantitative Insights into Microbial Ecology (QIIME2, version 2019.7) was used for the analysis of the samples (Bolyen et al., 2019). Sequences were clustered into operational taxonomic units (OTUs) using the Uclust algorithm at 97% sequence similarity (Edgar, 2010). The taxonomies were assigned to the OTUs by aligning the reads against the Greengenes Database (version 13_8) (McDonald et al., 2012) based on a threshold of 97% sequence similarity. The functional metagenomic profile and metagenomic content of the samples were predicted from the 16S rRNA profiles, and KEGG pathway functions were categorized at level 3 using the phylogenetic investigation of communities by reconstruction of unobserved states (PICRUSt) tool (Langille et al., 2013) and visualized using STAMP (Software package for analyzing taxonomic or metabolic profiles) tool.

### Statistical Analysis

Weighted and unweighted UniFrac distances analysis of the samples was done from the normalized OTU table. Alpha-Diversity values of the samples were calculated by the function using the Shannon method in QIIME2 and R to obtain the observed faith-pd, Shannon entropy, observed features, and pielou-evenness (Kotoky and Pandey, 2020).

## Results

### Physicochemical Analysis of the Samples

The physicochemical parameters of water samples are mentioned in Table 1. The temperature of the sample Hot-spring2 was comparatively higher, but its turbidity was lesser than Hot-spring1. The pH was recorded higher with temperature range, variable conductivity, and salinity. Dissolved oxygen (DO), Biological oxygen demand (BOD), and chemical oxygen demand (COD) were measured to understand the level of oxygen concentration. Both samples Hot-spring1 and Hot-spring2 had shown BOD in the normal range but the COD of sample Hot-spring2 was found to be much higher than Hot-spring1 demonstrating the presence of more organics in the water. The Total Dissolved Solids (TDS) was also under the good range for both the samples. The hot springs were chosen for the study due to their different conditions of pH and temperature. Both the hot springs were found to be alkaline but with different temperatures (55 and 65°C). The sample Hot-spring2 had a relatively high concentration of salts than Hot-spring1.

**Table 1:** Physicochemical analysis on water sample.

**Sample**	**Temperature** ** (area) ^**°**^C**	**Temperature** ** (sample) ^**°**^C**	**pH**	**Humidity** ** %**	**Electrical** ** Conductivity** ** ms**	**Turbidity** ** NTU**	**DO** ** mg/L**	**BOD** ** mg/ml**	**COD** ** mg/L**	**TDS** ** (ppm)**	**NaCl** ** Conc**
Hot Spring 1	25.4	50-55	8.5	34	791	7.12	9	187	389	412	0.02
Hot Spring 2	33.2	60-65	9.5	58	827	5.96	17.1	120	1144	376	0.02

### Isolation of Thermophiles and Analysis of the Activity of Enzymes

From the two hot spring samples, 28 thermophilic bacterial isolates were isolated. The isolated bacterial strains were analyzed for the production of different industrial enzymes such as protease, lipase, amylase, cellulase, pectinase, xylanase, gelatinase, and lecithinase. From the isolates, seven isolates, including–CAP1, CAP3, CAP7, BAC18, BAC23, BAC26, BAC28 showed excellent potential for enzyme production. Twenty-three isolates (82%) produced protease, 27 isolates (96%) produced lipase, 27 isolates produced amylase, 26 isolates (92%) produced cellulase, 19 isolates (67%) produced pectinase, 13 isolates (46%) produce gelatinase and 19 isolates (67%) could produce lecithinase. The study showed, all isolated thermophilic bacteria showed enzyme activities for at least three enzymes.

**Figure 1:**
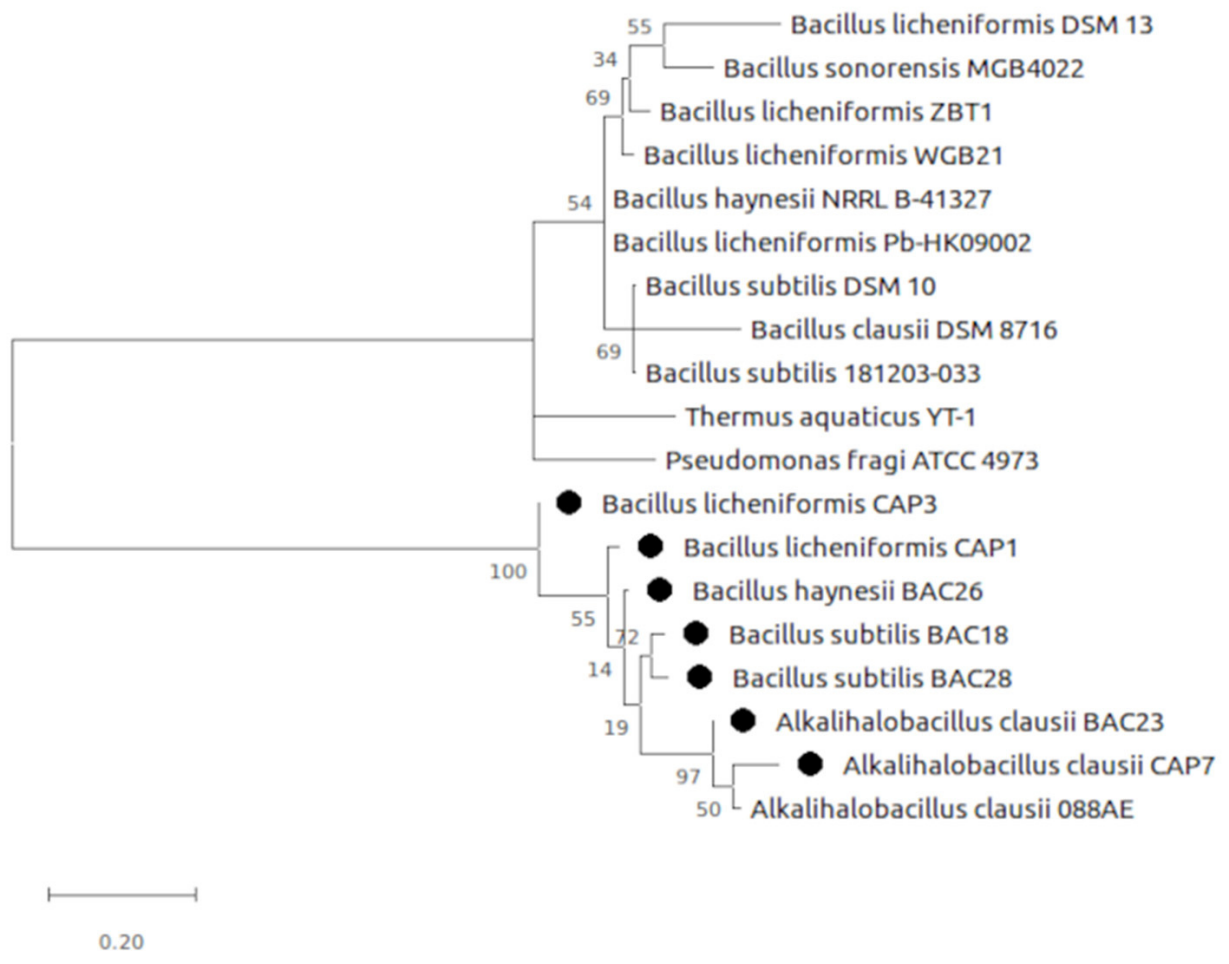
Evolutionary analysis by Maximum Likelihood method. The evolutionary history was inferred by using the Maximum Likelihood method and Tamura-Nei model (Tamura and Nei, 1993). The tree is drawn to scale, with branch lengths measured in the number of substitutions per site. Evolutionary analyses were conducted in MEGA X (Kumar et al., 2018).

The selected bacterial isolates were characterized and identified as *Bacillus licheniformis* CAP1, *Bacillus licheniformis* CAP3, *Alkalihalobacillus clausii* CAP7, *Bacillus subtilis* BAC18, *Alkalihalobacillus clausii* BAC23, *Bacillus haynesii* BAC26, and *Bacillus subtilis* BAC28. The phylogenetic analysis of the isolates placed the organisms in at distinct branches of the dendogram (Figure 1). The quantitative analysis of the enzymes revealed that *B. licheniformis* CAP1 produces the highest amount of protease (62.14 U/ml) at given ambient temperature and pH, also showed good production of cellulase and amylase, 60.11 U/ml and 59.83 U/ml, respectively. Cellulase activity was also found to be maximum for isolate CAP1. *Bacillus licheniformis* CAP3 produced the highest amount of amylase (61.26 U/ml) and *Alkalihalobacillus clausii* BAC23 produced the highest activity of lipase (51.25 U/ml). The activity of lecithinase was found to be less than other enzymes and was in the range of 27–37 U/ml. Gelatinase activity was observed highest in *Bacillus haynesii* BAC26 (51.23 U/ml) while other isolates showed less production of the enzyme at given ambient temperature and pH. Conclusively, all the seven isolates were observed in amylase production ranges from 49 to 61 U/ml, lipase in the range 33–51 U/ml, cellulase in the range 38–60 U/ml, protease in 31–62 U/ml, and gelatinase 24–51 U/ml (Table 2).

**Table 2:** Production of enzyme at ambient temperature (T_opt_) and pH (pH_opt_).

**Isolates**	**T_**OP*t***_^**°**^C**	**pH_**OPT**_**	**Amylase** ** U ml^**−1**^**	**Lipase** ** U ml^**−1**^**	**Cellulase** ** U ml^**−1**^**	**Protease** ** U ml^**−1**^**	**Lecithinase** ** U ml^**−1**^**	**Gelatinase** ** U ml^**−1**^**
*Bacillus licheniformis* CAP1	55	8.5	59.83	43.07	60.11	62.14	27.15	26.23
*Bacillus licheniformis* CAP3	55	8.5	61.26	36.10	39.26	36.28	31.19	28.21
*Alkalihalobacillus clausii* CAP7	55	8.5	61.19	44.23	43.28	53.12	28.54	33.23
*Bacillus subtilis* BAC18	65	9.5	58.17	47.36	51.18	31.15	33.33	35.71
*Alkalihalobacillus clausii* BAC23	65	9.5	55.23	51.25	38.20	42.18	37.39	41.26
*Bacillus haynesii* BAC26	65	9.5	51.85	33.09	48.51	51.67	28.19	51.23
*Bacillus subtilis* BAC28	65	9.5	49.87	38.67	51.19	45.83	29.24	24.18

### Composition of Microbial Community

**Figure 2:**
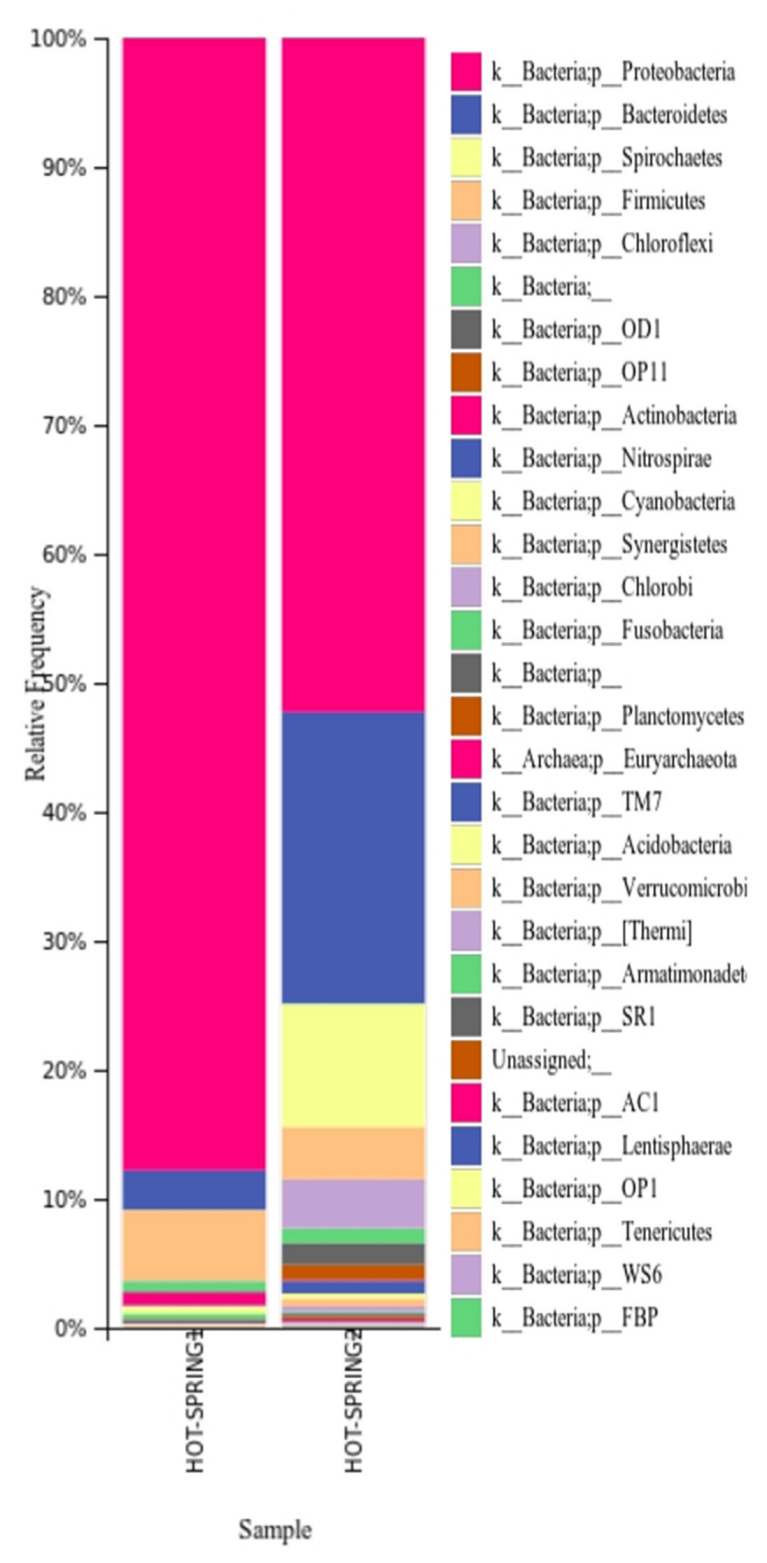
Distribution of different taxa in the hot-spring samples analyzed at phylum level.

The taxonomic analysis of the microbiome of the hot springs showed a predominance of bacteria and relatively very less proportion of archaea. In both the samples, the phylum Proteobacteria was found to be more abundant as plotted (Figure 2) however, the abundance of proteobacteria in hot-spring 1 (~88% of total abundance) was more than the hot-spring 2 (~52%). The phylum Bacteroides was found to be the second most abundant group (~10–22%) in the hot-springs but very different from each other. The other phyla with more abundance were Spirocheates, Firmicutes, Chloroflexi, etc.

The analysis at the genus level showed a very high abundance of an unknown genus from family commamonadaceae in both samples. The alpha-diversity analysis was done on the processed data and the faith_pd, shannon_entropy, and pielou_evenness of the samples have been calculated. The analysis showed that the hot-spring 1 was more diverse and had diversity richness (Table 3). The observed diversity in the microbiome was then compared with the alpha-diversity of different hot-spring samples of a different part of the world (Table 4). Which showed that the pH and geographical location of the hot-springs play a very crucial role in shaping their microbial diversity.

**Table 3:** Alpha-diversity indices of the samples.

**Samples**	**faith_pd**	**shannon_entropy**	**pielou_evenness**
Hot-spring 1	72.33	4	0.44
Hot-spring 2	48.78	5.24	0.65

**Table 4:** Alpha-diversity of the microbiome of the hot-springs of different part of world.

**Sample** ** origin**	**Temperature** **^**°**^C**	**pH**	**Number of** ** distinct species**	**References**
China	65	7	457.73	Menzel et al., 2015
Colombia	29	2.7	467.61	Jiménez et al., 2012
Iceland	85–90	5	196.14	MGRAST ID: mgm4530143.3
Italy	76	3	86.12	MGRAST ID: mgm4529716.3
Russia	61–64	5.8–6	615.97	MGRAST ID: 4544453.3
Spain	76	8.2	330.87	Lopez-Lopez et al., 2015
India	55–65	8.5–9.5	410.5	This study

### Functional Analysis of the Microbiome of the Hot Springs

The functional analysis of the microbiome revealed different features related to several functions categorized at different KEGG (Kyoto Encyclopedia of Genes and Genomes) level annotations. KEGG system analysis at level 1, significant differences in the abundance of genes for the different subsystems between the two samples. In hot-spring 1 the attributes related to cellular processes and environmental information processing were found to be significantly higher than hot-spring 2. However, the hot-spring 2 sample had greater attributes for genetic information, metabolism, and human diseases. The most of predicted protein sequences were associated with different functions related to metabolism (48–52%), environmental information processing (13–18%), genetic information processing (12–16%), and cellular processes (2–4%).

The functional prediction and annotation of the microbiomes at KEGG level 2, revealed a predominance of genes belonging to carbohydrate metabolism, amino acid metabolism, and membrane transport. The clustering of the attributes was done using the UPGMA method with a threshold of 0.75, which clustered the similar abundant attributes in both samples.

**Figure 3:**
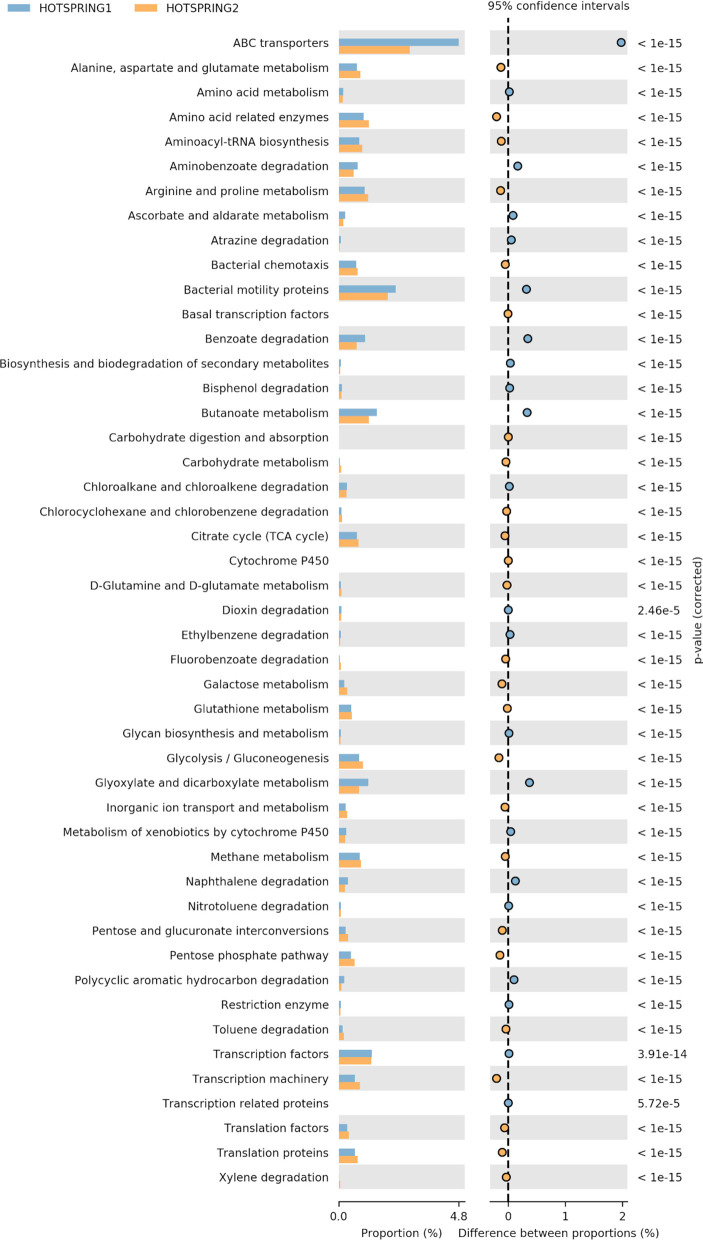
Variation in abundance of selected attributes between the hot-spring samples annotated at KEGG level 3.

The functional prediction and annotation of the microbiomes at KEGG level 3 annotated the sequences into 279 active features that showed variation in abundance between the samples. From the active features, 39 features were selected for analysis related to carbohydrate, protein, and fat metabolism and attributes related to the degradation of xenobiotic compounds. The functional analysis showed hot-spring 1 as more diverse functionally and have more abundance of attributes related to ABC transporters, amino acid metabolism, and genes for degradation of xenobiotic compound degradation. However, hot-spring 2 showed more abundance genes of metabolism of carbohydrates, lipids, and proteins, showing a greater abundance of functions related to industrial enzymes (Figure 3). The abundance of pathways related to ABC transporter (ko02010), bacterial motility proteins (ko02030), benzoate degradation (ko00362), starch and sucrose metabolism (ko00500), beta alanine metabolism (ko00410) were found to be significantly different between the two samples. On the other hand, the KEGG pathways related to calcium signaling pathway (ko04020), lipid biosynthesis process (ko00061), glycan biosynthesis (ko00510) etc. were found to be significantly low in abundance and less diverse between the samples.

### Data Availability

The metagenomic sequences of the samples were deposited in NCBI, at Sequence Read Archive (SRA) under the accession number SRP13358614 and SRP13358615; Bioproject ID PRJNA688206 and BioSample ID- SAMN17170341 (Hot-spring 1) and SAMN17170342 (Hot-spring 2). The 16s rDNA sequences of the selected bacterial isolates were submitted to NCBI Genbank under the accession numbers MW527298 (*Bacillus licheniformis* CAP1), MW527299 (*Alkalihalobacillus clausii* CAP7), MW527300 (*Bacillus subtilis* BAC18), MW527301 (*Bacillus licheniformis* CAP3), MW527302 (*Alkalihalobacillus clausii* BAC23), MW527303 (*Bacillus haynesii* BAC26), and MW527304 (*Bacillus subtilis* BAC28).

## Discussion

The hot springs are considered to be the source of untapped microbial diversity, that are a source of enzymes of industrial importance. Therefore, the microbial diversity of two hot springs has been characterized and the functional roles of the microbiome were predicted using the metagenomics approach. Further, seven potential isolates were cultured and found efficient for industrially potential enzymes, active at high temperatures. Several studies of hot spring environments have focused on the relationship between microbial communities and different environmental factors especially temperature, which is believed to be the main factor that drives the community structure (Skirnisdottir et al., 2000). However, different other factors like available organic carbon, total dissolved solids, salt concentration also play a crucial role in shaping the microbial community structure as the microbial diversity do not have a monotonic relationship with temperature, where different other environmental or spatial factors may also be responsible for determining the microbial community (Purcell et al., 2007). It has been reported that the microbial community structures were different in the low- and high-sulfide hot spring mats with the same temperature (Skirnisdottir et al., 2000). Moreover, the hyperthermophilic archaeal communities are different in various hot springs. Therefore, the environmental and spatial variables play an important role in shaping microbial community compositions in natural ecosystems (Zhang et al., 2018). Power et al. (2018) and Uribe-Lorio et al. (2019) have reported that pH has a strong influence on the microbial community structure, where the influence of temperature was significant only at values above 70°C. Purcell et al. (2007) also reported that the high temperature (75–90°C) and alkaline pH (7.5–9) were the most influencing factors shaping the microbial community of the hot springs of Thailand.

Hot springs are the main source of microbial diversity to find industrially important enzymes (Sahay et al., 2017). The thermostable enzymes are stable and active even at temperatures higher than the optimal growth temperature showing potential for numerous industrial applications. Moreover, these enzymes have been reported to be more stable also against many solvents, detergents, and acidic and alkaline pH (Bhalla et al., 2013). Mohammad et al. (2017) also reported 10 thermophilic bacteria isolated from Jordanian Hot-spring could produce a wide range of thermostable enzymes leading to potential applications of the bio catalyzed processes in harsh conditions. Different thermostable bacterial enzymes like α-amylase, protease, and lipase have been used extensively in industrial processes. These thermophilic and hyperthermophilic enzymes are part of the enzyme category called extremozymes, which involve functions at extreme conditions like high salt levels, high alkaline conditions, or under extreme conditions of pressure or acidity (Vieille and Zeikus, 2001). The stability of the enzymes depends on the thermodynamic and kinetic stabilities. In the present study, the activity of the enzymes at ambient temperature was found to be high and have very good potential to be used for production.

The culture depended analysis of the bacterial population of the hot springs led to the isolation of 28 bacterial isolates that showed good enzyme activity of industrial importance. From the isolates, seven isolates were identified as having all the enzyme activity and were from phyla firmicutes. However, a culture-independent analysis of the microbiome of the hot-springs showed many unidentified classes and families, which are still left to be investigated. The taxonomic identification of the microbiome was done using Greengene classifier revealing many known and unknown bacterial taxa, and proteobacteria as most abundant. Different other studies also reported proteobacteria as dominant taxa in the hot springs with moderately high and very high temperatures (44–110°C) at various geographical locations, including India (Chan et al., 2015; Ghelani et al., 2015). Different earlier studies have suggested a decrease in diversity of the microbial community with increasing environmental temperature (Mathur et al., 2010; Valverde et al., 2012). Interestingly, the taxonomical analysis showed the hot springs has a diverse and different pattern of abundance although both have different temperature, pH, and the influx of organic material. Thus, it can be assumed that the community structure is largely determined by a combination of environmental parameters, rather than geographical distance.

The taxonomic and functional study of the microbial ecology in the hot-springs showed the influence of environmental factors like temperature, pH on the microbiome that boost the metabolism pattern and enhance the stress biology. The microbiome contains the functional groups that perform various metabolic functions. The metabolism of methane was found to be higher in hot-spring 2 with higher pH and temperature. The presence of a large number of phylogenetically diverse, metabolically divergent groups indicates a balanced complex community, where each group occupies its environmental niche. The temperature of the hot-springs was found to be in the range of 55–65°C, different from each other. However, several studies reported that the temperature is not a unique determinant of microbial diversity and its function in the hot springs (Huang et al., 2011; Wang et al., 2013). Importantly, the pH of the springs was found in the range of moderate alkaline (8.5–9.5). The higher pH ranges have been reported to significantly impact the biodiversity of certain biological niches leading to the association of different adapted microbial groups. As reported by Tyson et al. (2004), the acidic pH of mine drainage site in Iron Mountain, California, USA (pH 0.83, 42°C) led to the selection of a very simple community dominated by an extremophilic Leptospirillum and Ferroplasma. At alkaline pH range also, the effect is reported to be similar. Therefore, the microbiome of hot-spring 2 (pH 9.5) in the present study was found less diverse than hot-spring 1 (pH 8.5). It has been reported that alkaline hot springs with a lower temperature below 73°C are typically dominated by cyanobacteria (Pedersen and Miller, 2016). However, in contrast to that, the hot-springs of the present study the cyanobacteria phylum was not on the higher abundance side, instead, the phylum spirochaetes were found to be very high in abundance in the sample Hot-spring 2 which was not that abundant in sample Hot-spring 1. Therefore, the effect of pH also playing a very crucial role in microbiome function and taxonomy, where the effects are both direct and indirect.

Several previous studies have reported different type of microbial structure in alkaline hot springs. Lopez-Lopez et al. (2015) described the bacterial phyla Deinococcus-Thermus as the most dominant in a alkaline Hot Spring in Galicia (Spain), followed by Proteobacteria (13%), and Firmicutes (10%). The archaea phylum Thaumarchaeota (6%) was found to be most abundant. Similarly, other studies also reported high occurrence of Thaumarchaeota in the archaeal fraction in alkaline springs from Kamchatka and China (Huang et al., 2011; Wemheuer et al., 2013). Menzel et al. (2015) reported that relative abundance of Archaea in hot springs is higher in low pH and high temperature environments. However, in the present study, at higher pH and temperatures very low abundance of archaea was observed. Interestingly, it has been reported that the most common substrate in alkaline hot-spring is hydrogen and sulfur (Horikoshi, 1999). These alkaliphilic microbes have adapted to such conditions through different mechanisms including the presence of cytoplasmic polyamines with charged amino acids. In *Bacillus* spp., in addition to peptidoglycan, there are acidic compounds such as galacturonic acid, gluconic acid, glutamic acid, aspartic acid, and phosphoric acid that act as buffers to the alkaline environment, allowing uptake of hydronium ions and exclusion of hydroxide ions (Horikoshi, 1999).

## Conclusion

The culture-dependent analysis of the water samples of the hot-springs led to the isolation of several bacterial strains having good enzymatic activities with significant industrial importance. The culture-independent analysis showed that the taxonomical and functional diversity of the hot springs were distinct and is possibly shaped by temperature, pH, and organic materials. The study showed the presence of different unassigned bacterial taxa with great abundance which indicates the potential of novel genera or phylotypes. Different taxa were found to be more prominent in higher temperature than others and it was observed that multiple factors like pH, salinity also play a great role in shaping a microbiome. The functional analysis of the microbiomes revealed that most of the genes are associated with functions related to metabolism and environmental information processing. The analysis showed the presence of metabolic and biosynthesis pathways of different primary substrates including carbohydrates, fats, proteins etc. which display its industrial importance. The microbiome study showed that the hotspring 1 with low temperature and pH was more diverse taxonomically and functionally.

## Data Availability Statement

The datasets presented in this study can be found in online repositories. The names of the repository/repositories and accession number(s) can be found in the article/supplementary material.

## Author Contributions

KC and SP did the sample collection and screening of the enzymes and wrote the first draft of the manuscript. RK did the metagenome analysis of the samples and wrote the second draft of the manuscript. ASri, AT, PR, and ASha did the characterization and shared ideas. PP did the conceptualization and revised the manuscript. All authors contributed to the article and approved the submitted version.

## Conflict of Interest

The authors declare that the research was conducted in the absence of any commercial or financial relationships that could be construed as a potential conflict of interest.
